# Analysis of heart rate variability in critical septic patients using a smartphone application: a prospective cohort study

**DOI:** 10.3389/fmed.2026.1877291

**Published:** 2026-08-11

**Authors:** João Raphael Zanlorensi Glir, Marcelo Mazza do Nascimento, Hygor Trombetta, Igor Alexandre Cortês de Menezes

**Affiliations:** 1 IntensiveCare Unit, Hospital Universitário Evangélico Mackenzie, Curitiba, Paraná, Brazil; 2 Department of Internal Medicine, Federal University of Paraná, Curitiba, Paraná, Brazil; 3 Internal Medicine and Health Sciences, Federal University of Paraná, Curitiba, Paraná, Brazil; 4 Intensive Care Unit, Hospital de Clínicas, Federal University of Paraná, Curitiba, Paraná, Brazil

**Keywords:** autonomic nervous system, heart rate variability, intensive care unit, mobile health, mortality, sepsis, wearable device

## Abstract

**Introduction:**

Heart rate variability (HRV) reflects autonomic nervous system modulation and has been associated with mortality in sepsis. However, in the intensive care unit (ICU), HRV assessment with traditional equipment limits its use. This study aimed to investigate the association between HRV measured by a mobile application and mortality in septic patients.

**Methods:**

This is a prospective observational cohort study conducted in two ICUs in Curitiba, Brazil, between July 2024 and March 2026, including septic patients (≥18 years) within 24 h of sepsis diagnosis. HRV, assessed using the standard deviation of NN intervals (SDNN), was measured through a heart rate monitor strap for 5 min over 3 consecutive days; clinical and hemodynamic data were also collected. Pregnancy, patients in exclusive palliative care, skin lesions that could interfere with the cardiac sensor, and decompensated irregular heart rhythm signs during data collection were excluded. The primary outcome was 28-day mortality.

**Results:**

A total of 112 septic patients were included, with an overall 28-day mortality of 49%. Non-survivors had a significantly higher APACHE II (30 vs. 22, *p* < 0,001), and SDNN values were also significantly lower in non-survivors at 72 h (9.4 ms vs 11.3 ms; *p* = 0,027). Additionally, in multivariable analysis, female sex, age, SOFA score, capillary refill time (CRT), and SDNN < 5 ms were independently associated with mortality.

**Conclusion:**

HRV assessed by a mobile application demonstrated a significant association with 28-day mortality in septic patients, with lower SDNN values at 72 h. This method might facilitate the wider implementation of HRV monitoring in resource-limited ICUs to stratify mortality in septic patients.

## Introduction

Sepsis is a syndrome secondary to an infection that leads to life-threatening organ dysfunction due to a dysregulated host response (1). Despite improvements in sepsis diagnosis and treatment, the mortality still represents around 30% of total global deaths, particularly in low- and middle-income countries where it can represent up to 55% (2). Initial sepsis management includes quickly identification, antibiotic administration, and precise hemodynamics compensation with fluids and vasoactive drugs to restore perfusion (3, 4).

Despite numerous predictive scores, Acute Physiology and Chronic Health disease Classification System II (APACHE II) (5), Sepsis-related Organ Failure Assessment (SOFA) (6, 7), Simplified Acute Physiology Score III (SAPS-3) (8), and various macro-microcirculatory goals, mean arterial pressure (MAP), cardiac output (CO), Lactate, CRT, tissue perfusion pressure (TPP), critical closing pressure (Pcc), peripheral perfusion index (Ppi) (9–11). None of these incorporates autonomic nervous modulation, which can be assessed by HRV (12).

HRV is regulated by the autonomic nervous system (ANS). The parasympathetic nervous system (PNS) decreases heart rate through the release of acetylcholine (ACh) via the vagus nerve, which innervates the sinoatrial and atrioventricular node, and atrial myocardium. In contrast, the sympathetic nervous system (SNS) increases heart rate and myocardial contractility by releasing catecholamines in the blood (13, 14). Over the past six decades, studies evaluating the reduction in HRV and changes in RR interval in the electrocardiograph (ECG) or Holter (13, 14) have demonstrated an association with poor survival outcomes in several conditions, such as decompensated heart failure, myocardial infarction, severe brain injury, and sepsis (15, 16).

In sepsis, multiple HRV parameters are reduced. One of the most common and highly correlated with other domains is the time domain variable, SDNN, which is independently associated with mortality in adults and pediatric populations (17–20).

Despite being widely studied, HRV assessment in intensive care remains challenging due to the limited availability of equipment and trained professionals to interpret the data. However, parameters such as SDNN can now be evaluated using smartphone applications integrated with a cardiac chest-strap after studies in healthy patients (23). This approach may enhance usability compared with devices such as Holter monitors, making it particularly useful in low- and middle-income country settings.

This study aimed to investigate the association between HRV and mortality using a smartphone application integrated with a cardiac chest-strap in ICU septic patients.

## Materials and methods

### Study design, setting & participants

This prospective observational cohort study was conducted in three intensive care units at the Hospital de Clínicas, Federal University of Paraná, and Hospital Universitário Evangélico Mackenzie, between July 2024 and March 2026 in Curitiba, Brazil. Written informed consent was obtained from all participants or their legal representatives. The study was approved by the Human Research Ethics Committees of the Hospital de Clínicas, Federal University of Paraná, and Hospital Universitário Evangélico Mackenzie (CAAE: 71345923.2.0000.0096 and CAAE: 71345923.2.3001.0103). The study was also duly registered on the REBEC platform (Brazilian Registry of Clinical Trials number RBR-4xzqtkv). All research procedures adhered to the ethical standards of the Declaration of Helsinki.

The study included adult patients (aged ≥18 years) with sepsis admitted to the ICU or within 24 h after sepsis onset. Patients previously admitted to the ICU for other indications were also eligible. Exclusion criteria included pregnancy, exclusive palliative care, skin lesions that could interfere with the cardiac sensor placement, unstable arrhythmias during data collection, and those who did not sign informed consent.

### Clinical definitions

#### Sepsis

Sepsis was defined according to the most recent consensus criteria (21). This syndrome is defined by the presence of infection combined with an acute change in the SOFA score of two or more points. Septic shock remains a subset of sepsis cases wherein, despite adequate resuscitation, patients have hypotension requiring vasopressors to maintain MAP above 65 mmHg and have an elevated serum lactate concentration ≥2 mmol/L.

### Heart rate variability

There are three major methods of measuring HRV (17). In this present study, the Time Domain Method was used because it is considered the easiest to evaluate through the heart rate. The intervals between successive QRS complexes are called normal-to-normal (NN) (15). Heart rate variability was quantified using SDNN, defined as the standard deviation of all normal-to-normal RR intervals during the ECG recording period, which reflects overall autonomic modulation (13).

### Study protocol

All patients were treated according to Surviving Sepsis Campaign (SSC) guidelines: 30 ml/kg of ringer lactate or crystalloid fluid was administered at the discretion of the patients' physicians over the first h of sepsis diagnosis, and if MAP remained < 65 mmHg, norepinephrine or a combination of vasopressors were used to maintain MAP ≥65 mmHg. Patients were followed from sepsis diagnosis until hospital discharge or 28 days, whichever occurred first. Survivors included patients who remained alive in the hospital and those discharged alive before day 28.

**Figure 1:**
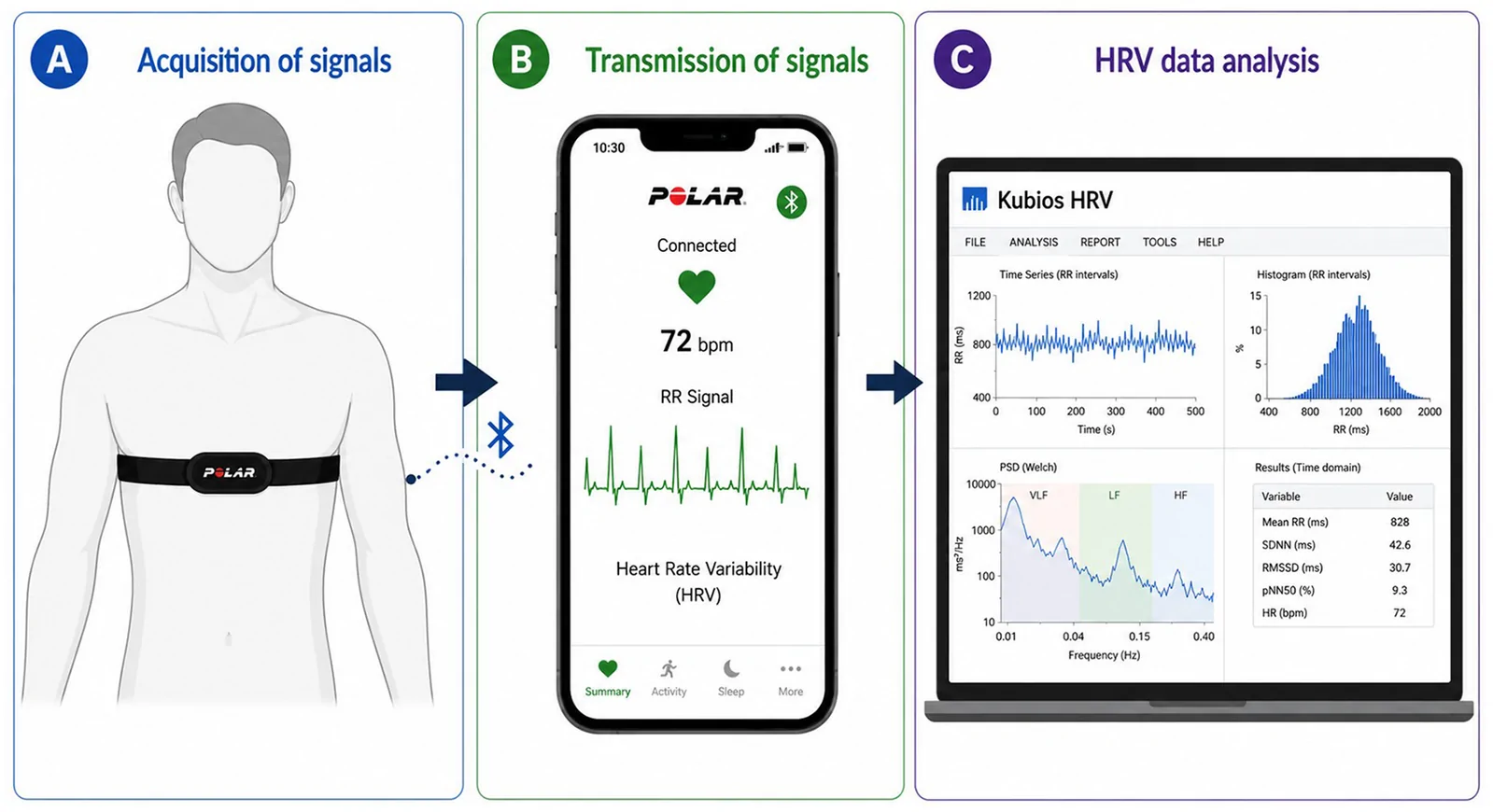
Workflow of heart rate variability (HRV) acquisition and analysis. **(A)** Signal acquisition using a chest-strap Polar heart rate monitor positioned around patient thorax. **(B)** Transmission and capture of RR interval signals via a smartphone application using Bluetooth connectivity. **(C)** Processing and analysis of HRV SDNN data using Kubios HRV software.

Demographic characteristics, comorbidities, the source of infection at admission, medication for chronic diseases, APACHE II, and SOFA scores were obtained. Patients were enrolled within 24 h of ICU admission with sepsis, and 24 h after the onset of sepsis in patients previously admitted for other causes. Patients were followed through day-3 in the intensive care unit, with laboratory data collected per institutional protocols (lactate, creatinine, urea, bilirubin, C-reactive protein), clinical data (respiratory rate, CRT, vasoactive drugs use, sedatives, and medications that may interfere with heart rate) and daily SOFA score. Heart rate was measured using a Polar H10^®^ heart rate monitor with patients in the supine position for 5 min during data collection (22, 23). The data were imported into Kubios HRV Scientific Software^®^ version 4.2.0, where the SDNN metric was automatically calculated (24), Figure 1.

### Clinical outcomes

The primary outcome was 28-day inpatient mortality in septic patients. For primary analysis, the association between SDNN measured on Day-1 to Day-3 after sepsis diagnosis and mortality was evaluated. Secondary analyses compared SDNN with clinical (APACHE II, SOFA) and hemodynamic/perfusion variables (Lactate, CRT, organ dysfunction support) to predict 28-day inpatient mortality.

### Statistical analysis

The Shapiro-Wilk test was used to assess normality. Parametric data were described as mean ± standard deviation, while non-parametric data were presented as median and interquartile range. Categorical variables were expressed as percentages. Patients were stratified into survivor and non-survivor groups. Continuous variables were compared using Student's t-test or Mann-Whitney U test, as appropriate based on distribution. Categorical variables were compared using Fisher's exact test.

For univariable and multivariable analysis of risk factors associated with mortality, binary logistic regression models were fitted using stepwise backward selection. Based on the number of outcome events, a maximum of 6 independent variables could be included in the multivariable model. Variables with the greatest statistical significance in univariable analysis were prioritized for inclusion. Strongly correlated or collinear variables were not included simultaneously. Although APACHE II and SOFA scores showed moderate correlation (r = 0.57) without meeting formal collinearity thresholds, they were not included together in the multivariable model because they represent overlapping dimensions of illness severity and to avoid overfitting with the limited number of events. The significance of variables in the final model was assessed using the Wald test, and associations were expressed as odds ratios (OR) with 95% confidence intervals (95% CI).

To determine a cutoff point for SDNN associated with death, an ROC curve was performed, with estimation of the area under the curve (AUC) and definition of the optimal cutoff point using the Youden index. To compare the groups regarding the evolution of SDNN over the 3 days, a linear mixed model for repeated measures was fitted with the logarithm of SDNN as the response variable, due to the right skewness in the distribution. Initially, the model included as fixed effects the groups (survivors and non-survivors), time of assessment (Day-1, Day-2, and Day-3), and the interaction between group and time, in addition to a random intercept for intraindividual correlation between repeated measures. As no significant interaction between group and time was observed (χ^2^(2) = 2.30;*p* = 0.317), a reduced model was fitted without the interaction term to estimate the main effects of group and time. This approach allowed the inclusion of patients with incomplete measures during follow-up. *P*-values < 0.05 were considered statistically significant. Statistical analyses were performed using Stata/SE version 14.1 (StataCorp LP, College Station, TX, USA) and GraphPad Prism 6.

The sample size was calculated *a priori* based on a pilot study of 27 septic patients. The survivor group had a mean SDNN of 1.007 with a standard deviation of 0.400 on the first day of sepsis. Considering that the sample mortality was approximately 29% and that the non-survivor group had a mean SDNN 50% lower (19), a sample of 99 patients was estimated to detect the difference between groups with 80% power and a type I error of 5%. Due to the non-parametric distribution of the data, the total sample size was increased by 15% (25), resulting in a final sample size of 115 patients.

This study followed STROBE guidelines for reporting results.

## Results

**Figure 2:**
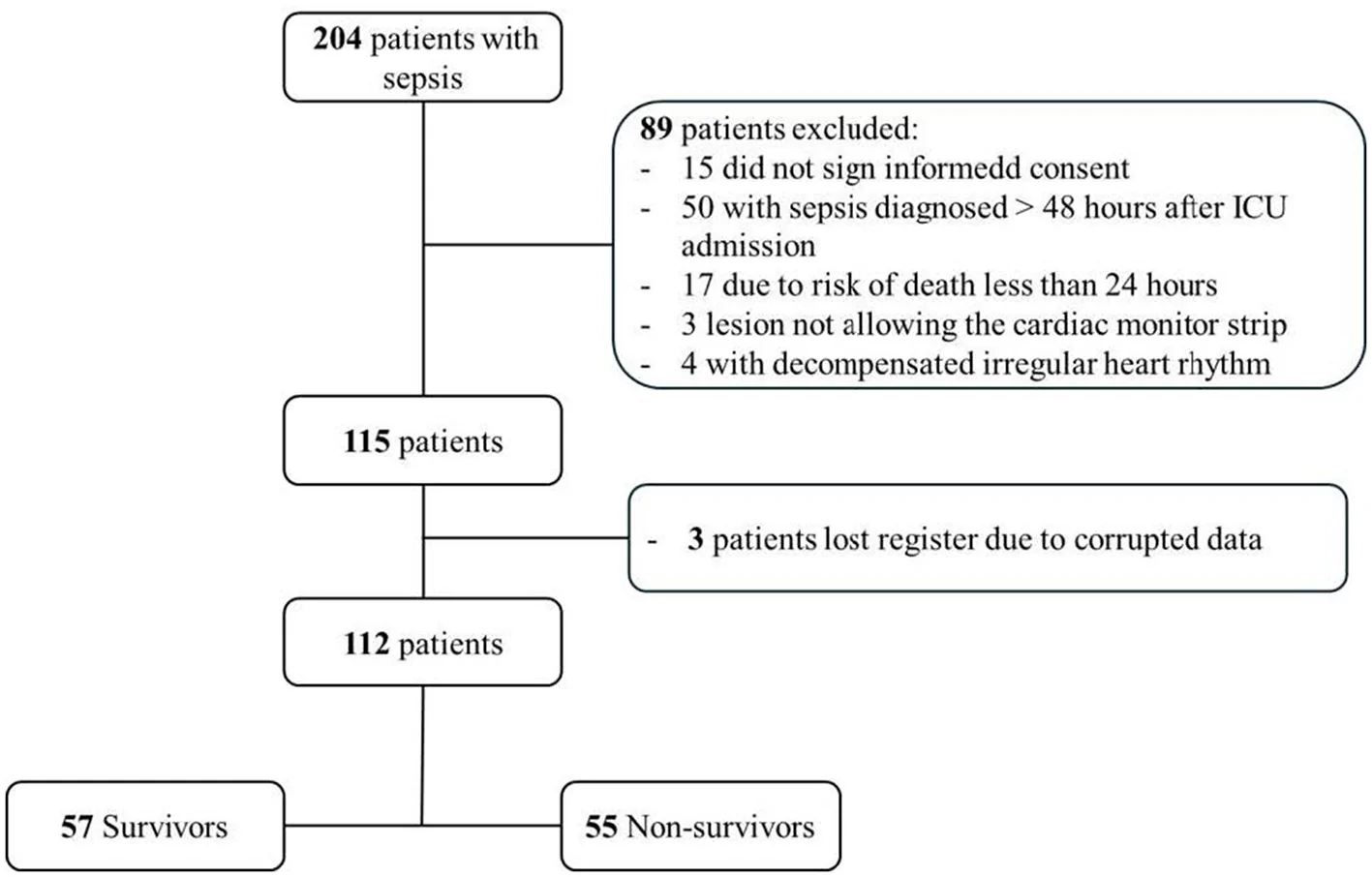
Flowchart of patient enrollment and follow-up.

**Figure 3:**
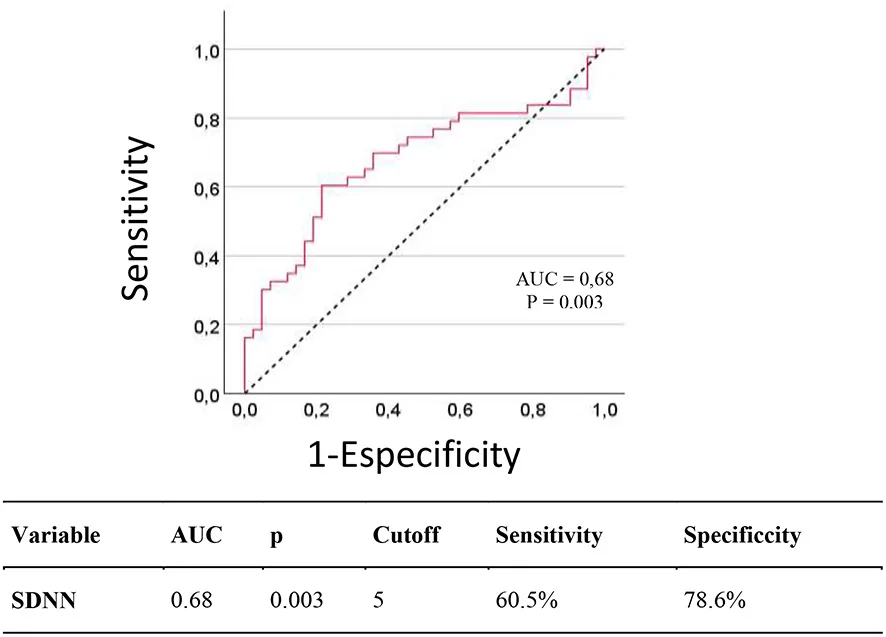
ROC curve and day-3 SDNN predicting 28-day impatient mortality.

In this cohort, 115 patients with sepsis were enrolled and underwent HRV assessment. As shown in Figures 2, 3 patients were excluded due to corrupted recordings, resulting in 112 patients in the final analysis. Overall 28-day mortality was 49% (55 of 112 patients).

The baseline data are presented in Table 1. Age, gender, and hypertension differed significantly between survivors and non-survivors. APACHE II scores were particularly high in both groups (22 in survivors and 30 in non-survivors) and statistically different, as was the SOFA score in the 3 days. The source of infection did not differ between groups.

**Table 1:** Baseline characteristics of survivors and non-survivors at 28 days.

Variables		Survivors (*n* = 57; 50.9%)	Non-survivors (*n* = 55; 49.1%)	*P*
Age (years)^*^		55.7; 60 (47–67)	67.1; 67 (61–75)	< 0.001
Female		21 (36.8%)	24 (61.8%)	0.009
Underlying disease *n*°(%)				
Hypertension		28 (49.1%)	39 (70.9%)	0.020
Diabetes		12 (21.1%)	15 (27.3%)	0.443
Insulin-depedent diabetes		5 (8.8%)	11 (20.0%)	0.098
Heart Failure		10 (17.5%)	10 (18.2%)	0.930
Coronary artery disease		5 (8.8%)	7 (12.7%)	0.501
Arrhythmia		5 (8.8%)	7 (12.7%)	0.501
Chronic kidney disease		14 (24.6%)	12 (21.8%)	0.731
Imunossupression		11 (19.3%)	8 (14.5%)	0.504
Cancer		14 (24.6%)	14 (25.5%)	0.913
Lung disease		10 (17.5%)	15 (27.3%)	0.219
Cerebrovascular disease^**^		4 (7.0%)	1 (1.8%)	-
Cirrhosis		6 (10.5%)	6 (10.9%)	0.948
Others		21 (36.8)	14 (25.5%)	0.196
APACHE II^*^		21.8; 22 (14–26)	29.9; 31 (22–38)	< 0.001
SOFA^*^	Day-1	7.3; 7 (4–9)	10.1; 10 (7–14)	0.002
	Day-2	6.2; 5 (3–8)	9.6; 9 (6–13)	< 0.001
	Day-3	5.7; 5 (4–7)	10.3; 11 (7–14)	< 0.001
Source of infection *n*°(%)				
Respiratory tract		24 (42.1%)	32 (58.2%)	0.090
Intra-abdominal		9 (15.8%)	3 (5.5%)	0.090
Urinary tract		10 (17.5%)	9 (16.4%)	0.868
Central nervous system^**^		0 (0.0%)	1 (1.8%)	–
Soft tissue infection^**^		4 (7.0%)	3 (5.5%)	–
Bloodstream infection		6 (10.5%)	6 (10.9%)	0.948
Other source^**^		6 (10.5%)	3 (5.4%)	–

^*^Results expressed as mean, median (interquartile range). ^**^Variables with fewer than 10 cases, no statistical test was applied.

Regarding hemodynamic and clinical parameters in the non-survivors (Table 2), the mechanical ventilation was significantly higher, regardless of ventilatory mode. Additionally, respiratory rate (RR) and capillary refill time (CRT) were also significantly elevated during the final 2 days of the assessment. Lactate, another hemodynamic biomarker, did not differ significantly between groups on any of the 3 days, and sedation use was statistically significant only on day-2 and the use of norepinephrine on Day-3.

**Table 2:** Clinical and hemodynamic parameters during the first 3 ICU days, stratified by 28-day survival status.

Variables	Day	Mechanical Ventilaation Mode	Survivors (*n* = 57; 50.9%)		Non-survivors (*n* = 55; 49.1%)		*P*
			*n*	Results	*n*	Results	
Respiratory rate^*^	Day-1		57	19.3; 18 (16–22)	55	20.6; 20 (17–24)	0.170
	Day-2		54	17.8; 17 (14–20)	51	20.7; 20 (16–23)	0.006
	Day-3		41	17.3; 17 (15–19)	43	21.3; 20 (16–25)	0.002
Mechanical ventilation	Day-1	No	38	64.4%	21	35.6%	
		VCV/PCV	12	36.4%	21	63.6%	0.011
		PSV	7	35.0%	13	65.0%	0.025
	Day-2	No	40	67.8%	19	32.2%	
		VCV/PCV	7	28.0%	18	72.0%	0.001
		PSV	7	33.3%	14	66.7%	0.008
	Day-3	No	30	69.8%	13	30.2%	
		VCV/PCV	6	28.6%	15	71.4%	0.003
		PSV	6	28.6%	15	71.4%	0.003
Sedation	Day-1		14	24.6%	15	27.3%	0.743
	Day-2		8	14.8%	19	37.3%	0.011
	Day-3		7	16.7%	14	32.6%	0.094
Norepinephine	Day-1		28	49.1%	33	60.0%	0.249
	Day-2		20	37.0%	28	54.9%	0.068
	Day-3		11	26.2%	27	62.8%	< 0.001
Lactate (mmol/dL)^*^	Day-1		50	2.0; 1.7 (1.3–2.3)	53	2.7; 2.2 (1.8–2.7)	0.052
	Day-2		35	2.2; 2.0 (1.3–3)	44	2.5; 1.9 (1.5–3)	0.423
	Day-3		26	2.0; 1.7 (1.4–2.6)	34	2.4; 2.0 (1.5–2.8)	0.198
CRT (s)^*^	Day-1		55	2.7; 2.0 (1–3)	54	3.5; 2.0 (1–5)	0.279
	Day-2		52	1.9; 2.0 (1–2)	50	3.1; 2.0 (1–5)	0.006
	Day-3		42	1.7; 1.5 (1–2)	43	3.4; 2.0 (1–5)	0.006
SDNN (ms)^*^	Day-1		57	13.5; 7.7 (4.2–14.9)	55	12.1; 5.6 (3–13.6)	0.217
	Day-2		54	12.5; 6.9 (4.1–15.4)	51	11.7; 3.9 (2.2–20)	0.162
	Day-3		42	11.3; 7.8 (5.2−12.3)	43	9.4; 4.3 (2.3–8)	0.027
Log SDNN	Day-1		57	2.1; 2 (1.4–2.7)	55	1.9; 1.7 (1.1–2.6)	0.217
	Day-2		54	2.1; 1.9 (1.4–2.7)	51	1.8; 1.4 (0.8–3)	0.162
	Day-3		42	2.1; 2.1 (1.7–2.5)	43	1.6; 1.5 (0.9–2.1)	0.027

^*^Results expressed as mean, median (interquartile range). VCV, Volume Control Ventilation, PCV, Pressure Control Ventilation, PSV, Pressure Support Ventilation. CRT, Capillary refill time.

### Clinical outcome

SDNN was compared between survivors and non-survivors (Table 2). SDNN and logSDNN differed significantly between groups on Day-3 (*p* = 0.027), odds ratio (OR) 0.58; 95% IC 0.36–0.94, suggesting that a lower SDNN could be associated with a higher risk of death within 28 days of follow-up (Table 3). ROC curve analysis yielded an AUC of 0.68 (60.5% sensitivity and 78.6% specificity), which was performed and determined the optimal Day-3 SDNN value (cutoff) of 5ms for outcome prediction (Figure 3).

**Table 3:** Univariable logistic regression analysis of factors associated with 28-day inpatient mortality.

Variables	Day	Mechanical Ventilaation Mode	Univariable analysis	
			OR (CI 95%)	*P*
Age (years)			1.07 (1.03–1.11)	< 0.001
Gender (female)			2.78 (1.29–5.97)	0.009
Hypertension			2.53 (1.16–5.51)	0.020
Beta-adrenergic agonists			14 (1.74–112.6)	0.013
Statins			2.69 (1.12–6.45)	0.027
APACHE II			1.09 (1,04–1.13)	< 0.001
SOFA total	Day-1		1.16 (1.06–1.27)	0.002
	Day-2		1.19 (1.08–1.31)	< 0.001
	Day-3		1.34 (1.17–1.54)	< 0.001
Respiratory rate (RR)	Day-2		1.13 (1.04–1.23)	0.006
	Day-3		1.20 (1.07–1.34)	0.002
Mechanical ventilation	Day-1	VCV/PCV	3.17 (1.30–7.69)	0.011
	Day-1	PSV	3.36 (1.16–9.72)	0.025
	Day-2	VCV/PCV	5.41 (1.93–15.2)	0.001
	Day-2	PSV	4.21 (1.46–12.1)	0.008
	Day-3	VCV/PCV	5.77 (1.83–18.2)	0.003
	Day-3	PSV	5.77 (1.83–18.2)	0.003
Norepinephine	Day-3		4.76 (1.89–12.0)	< 0.001
Sedation	Day-2		3.41 (1.33–8.75)	0.011
CRT (s)	Day-2		1.48 (1.12–1.94)	0.006
	Day-3		1.65 (1.15–2.35)	0.006
Log(SDNN)	Day-3		0.58 (0.36–0.94)	0.027
SDNN < 5 ms	Day-3		4.89 (1.92–12.5)	< 0.001

APACHE II, acute physiology and chronic health evolution II; SOFA, sequential organ failure assessment, CRT, capillary refill time; LogSDNN, Logarithm of standard deviation of the NN intervals was used due to its asymmetric distribution.

Other factors associated with 28-day inpatient mortality identified by univariable logistic regression are listed in Table 3. For example: Age OR 1.07; 95% IC 1.03–1.11; APACHE II OR 1.09; 95% CI 1.04–1.13; SOFA at Day-2 and Day-3; norepinephrine uses on Day-3 OR 4.76; 95% IC 1.89–12.0 and SDNN < 5ms on Day-3 OR 4.89; 95% IC 1.92–12.5.

In multivariable logistic regression analysis (Table 4), age and female patients remained independent predictors of death in inpatients within 28 days. On the third day, respiratory rate, SOFA score, and CRT were also independent risk factors for death. Moreover, SDNN < 5ms on Day-3 was strongly associated with mortality (more than six-fold) within 28 days in this septic population after adjustment for confounders (*p* = 0.009; OR 6.55; 95% IC 1.59–27.1).

**Table 4:** Univariable and multivariable logistic regression analyses of factors associated with 28-day inpatient mortality.

Variables	Univariable analysis		Multivariable analysis	
	OR (CI 95%)	*P* ^*^	OR (CI 95%)	*P* ^*^
Age	1.07 (1.03–1.11)	< 0.001	1.06 (1.00–1.14)	0.068
Gender (female)	2.78 (1.29–5.97)	0.009	5.22 (1.29–21.1)	0.021
Respiratory rate (day-3)	1.20 (1.07–1.34)	0.002	1.22 (1.01–1.48)	0.043
SOFA (day-3)	1.34 (1.17–1.54)	< 0.001	1.29 (1.09–1.52)	0.003
CRT(s) (day-3)	1.65 (1.15–2.35)	0.006	1.73 (0.97–3.10)	0.063
SDNN < 5 ms (day-3)	4.89 (1.92–12.5)	< 0.001	6.55 (1.59–27.1)	0.009

^*^Wald test, *p* < 0, 05. Age, each additional year; Respiratory Rate at day-3, for each 1 respiratory movement increase; SOFA at day 3, sequential organ failure assessment, for each 1 point increase; CRT, capillary refill time at day 3, for each 1 s increase; SDNN at day 3, standard deviation of the NN intervals, if value under 5 ms.

**Figure 4:**
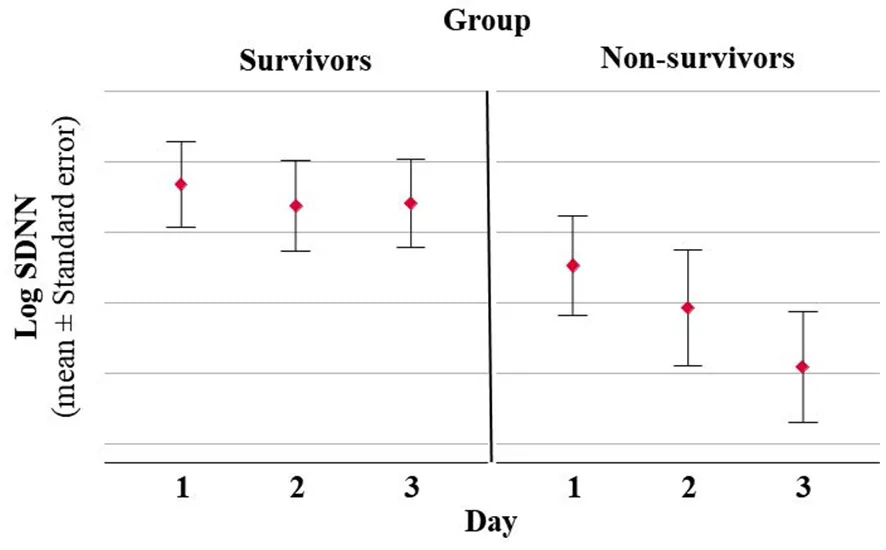
Temporal evolution of logSDNN (mean ± SE) during the first 3 ICU days in survivors and non-survivors.

In the reduced mixed-effects model—Figure 4, the non-survivor group demonstrated lower logSDNN values (coefficient = −0.338; IC 95% −0.640 to −0.036; *p* = 0.028), corresponding to approximately 29% lower mean SDNN values. Although there was a trend toward reduced logSDNN on Day-3 compared to Day-1, the result was not statistically significant (coefficient = −0.175; 95% CI: −0.381 to 0.032; *p* = 0.098).

## Discussion

HRV is a valuable tool in risk stratification, as well as providing information about the autonomic nervous system in both unselected critically ill patients (16) and especially in those with sepsis (19). To our knowledge, this is the first study to evaluate HRV in septic patients using a mobile application and a chest-monitor strap, demonstrating a significant association between lower SDNN values on day-3 and 28-day inpatient mortality.

There is a physiological rationale for this association. In sepsis, immune-inflammatory and vasoactive mediators compromise tissue homeostasis, resulting in dysoxia. This triggers the release of corticotropic hormones including corticotropin-releasing hormone (CRH), adrenocorticotropic hormone (ACTH), and cortisol, which activate autonomic nervous system responses through vagal afferent pathways (26). These pathways modulate both cardiac autonomic tone and neurohumoral actions. Acetylcholine (ACh), for example, attenuates the release of tumor necrosis factor (TNF), interleukin (IL)-1, IL-6, and IL-18, thereby reducing shock severity (16). These mechanisms explain why reduced HRV reflects diminished physiological capacity to match oxygen delivery with metabolic demands following sepsis-induced injury. Consistent with previous investigations, our findings demonstrate lower SDNN values in septic patients who did not survive (17–20, 27).

Despite this pathophysiological understanding, establishing standardized SDNN thresholds remains challenging. Studies in healthy Korean populations have found a mean SDNN value of 39.6 ms (28). In contrast, in a landmark study by Kleiger et al., SDNN < 50 ms was associated with a fivefold increase in mortality after myocardial infarction (29). In septic patients, SDNN values varied between survivors and non-survivor groups: 19 ms and 8.5 ms (19), to 9.4 ms and 5.6 ms in other cohorts (20). The average SDNN in this present study (13.5 ms for septic survivors on day 1 to 9.4 ms in non-survivors on the day 3) was consistent with previous findings. However, only at Day-3 did these values appear to be significantly different between the groups, an important temporal difference in our cohort.

Previous investigations identified significant HRV mortality associations within the first 24 h (18–20). Important methodological differences include the short-term measurement protocol (5 minutes) and the use of a cardiac chest-strap, for which comparable studies are limited. Nonetheless, Munoz et al. demonstrated that 2 min of measurement was sufficient for accuracy (30) and Schaffarczyk et al. demonstrated minimal bias in NN intervals when comparing the chest-strap used in this study with ECG for resting and recreational exercises (23). Furthermore, previous studies typically used Holter monitoring and ECG, which may capture nuances in ANS response, including circadian and respiratory influences (12, 18, 20), potentially explaining earlier detection in HRV. These temporal and methodological differences justify considering the sample characteristics.

Baseline population characteristics may have influenced temporal and HRV dynamics. The non-survivor group demonstrated unexpectedly higher female prevalence, with a fivefold increased risk of mortality within 28 days. In previous studies, women, especially those older than 50 years, typically demonstrated lower sepsis mortality risk and a lower HRV compared with men (31–33). Additionally, higher illness severity (mean APACHE II scores of 22 and 30 in survivor and non-survivor groups, respectively) compared with other studies indicates more pronounced organ dysfunction and greater autonomic nervous system impairment.

The combination of these factors (measurement methodology, sample characteristics, sex distribution, and illness severity) may have contributed to statistically significant SDNN differences emerging on Day-3 rather than Day-1. Interestingly, the non-survivor group demonstrated a trend toward a 29% average decrease in SDNN over 72 h. Ahmad et al. observed a similar pattern (25% decrease from baseline 35 h before sepsis onset) (27), suggesting a concordance in the physiological trajectories that supports our findings.

Regarding secondary analyses, SDNN demonstrated moderate performance (AUC 0.68) for predicting 28-day mortality. Notably, SDNN as a dynamic variable, showed a particularly strong association with 28-day mortality, especially at values below 5 ms (OR 6.55), compared with SOFA (OR 1.29) and CRT (OR 1.73) on Day-3. Zhang et al. demonstrated that combining HRV with SOFA and APACHE II scores could further improve discrimination (34). Previous studies by Singer et al. and Ait-Oufella et al. have already demonstrated associations between SOFA and CRT with mortality, respectively (21, 35). These three variables share a hemodynamic relationship: autonomic dysfunction compromises vascular regulation and reduces cardiac output, leading to tissue hypoperfusion, as evidenced by increased CRT (36). The consequence of hypoperfusion is organ dysfunction, expressed by an increase in SOFA. This pathophysiological cascade corroborates the importance of autonomic nervous system evaluation in septic patients. However, while these findings are promising, external validation is needed before widespread clinical implementation, and a bedside-accessible device is crucial for practical application.

The increasing use of wearable devices in healthcare services has demonstrated interesting capabilities for detecting clinical deterioration, predicting, and stratifying potentially life-threatening situations, especially in the ICU (37, 38). In this context, the present study utilized a cardiac chest-strap integrated with a smartphone application, a device previously validated as reliable for HRV detection. To our knowledge, this is the first application of such a device for HRV monitoring in critically ill septic patients. Acquisition of HRV and NN interval for SDNN analysis using Kubios Software proved feasible, reproducible, and accessible alternative, particularly in resource-limited settings such as low- and middle-income countries where Holter monitoring is not easily available.

However, technical challenges involving wearable devices warrant acknowledgment: signal acquisition was occasionally compromised by motion artifact and extreme body habitus, requiring repeated data collection, as well as signal instability during Bluetooth transmission between the chest-strap and smartphone application. These known technical factors (38) may contribute to measurement heterogeneity, but they do not invalidate the observed associations; they should be systematically addressed in subsequent investigations. Beyond the technical challenges, three methodological limitations warrant consideration. First, although the sample size was increased by 15% to account for non-parametric distribution, three corrupted recordings could not be recovered, which could have affected statistical power. Second, the multivariable analysis may carry residual confounding due to the limited number of variables included. Finally, the two-center design limits generalizability due to the heterogeneity of sepsis across populations.

Despite these limitations, the study demonstrated that HRV acquired via cardiac chest-strap using a smartphone application was significantly associated with 28-day inpatient mortality in septic ICU patients. Significant SDNN differences at Day-3 reflect complex interactions among autonomic dysfunction, population characteristics, disease severity, and measurement methodology. Wearable devices represent a potentially feasible option for autonomic monitoring in resource-limited critical care settings.

## Data Availability

The raw data supporting the conclusions of this article will be made available by the authors, without undue reservation.
